# Does capsaicin have therapeutic benefits in human colon adenocarcinoma? Selection of the most reliable dose via AgNOR

**DOI:** 10.3906/sag-2003-251

**Published:** 2020-06-23

**Authors:** Mustafa NİSARİ, Recep ERÖZ

**Affiliations:** 1 Department of Nutrition and Dietetics, Faculty of Health Science, Nuh Naci Yazgan University, Kayseri Turkey; 2 Department of Medical Genetics, Medical School, Düzce University, Düzce Turkey

**Keywords:** AgNOR, capsaicin, human colon adenocarcinoma, rDNA, caco-2 cell line

## Abstract

**Background/aim:**

To determine the effect of different doses of capsaicin on AgNOR protein synthesis in human colon adenocarcinoma derivate from colon cancer (Caco-2 cell).

**Materials and methods:**

In this experimental study, after the cultured of Caco-2 cell line, the cells are divided into 4 groups as control and different capsaicin exposed doses (25uµ, 50uµ, and 75uµ). Mean AgNOR number and total AgNOR area/nuclear area (TAA/NA) were calculated.

**Results:**

A significant differences were detected between control and capsaicin (50uµ) (P = 0.001), between control and capsaicin (75uµ) (P = 0.000), between capsaicin (25uµ) and capsaicin (50uµ) (P = 0.001) and between capsaicin (25uµ) and capsaicin (75uµ) (P = 0.000) for TAA/NA. Also, there were significant differences between control and capsaicin (50uµ) (P = 0.001), between control and capsaicin (75uµ) (P = 0.000), between capsaicin (25uµ) and capsaicin (50uµ) (P = 0.000) and between capsaicin (25uµ) and capsaicin (75uµ) (P = 0.000) for mean AgNOR number.**Conclusion:** A certain amount of capsaicin has a protective effect against colon adenocarcinoma and the dose concentrations are important for the most reliable treatment.

## 1. Introduction

As an important and major health problem, cancer is a major cause of human mortality after cardiovascular disease throughout all over the world [1,2]. Adenocarcinoma is among the most important tumors placed in the duodenum and duodena-jejunal junction and occurred around 30%–40% of all cancers of the small intestine [3]. Because of the increasing rate of cancer, different therapeutic approaches such as chemotherapy, radiation therapy, surgery, hormonal therapy, immunotherapy, targeted therapy are performed. Additionally, it is known that exercise and dietary phytochemical may significantly impact the prevalence of specific types of cancers. Phytochemical agents including alkaloids, polyphenols, carotenoids, and nitrogen compound naturally found in vegetables, fruits, grains, and other plant products. From the past to the present, medicinal plants have been traditionally used for the treatment of human disorders such as cancer and about 70% of antitumoral drugs are natural products or their derivatives [4,5].

Some proteins and ribosomal DNA (rDNA) that are transcriptionally active compose of nucleolar organizing regions (NORs) on chromosomes are argyrophilic features and converted the preribosomes in the nucleolus and mature ribosomes in the cytoplasm, respectively [6]. Different studies about the importance of the interphase AgNOR quantity in different cells were done [7–25]. To the best of our knowledge, there is no study about the antitumoral effects of different capsaicin dose in human colon adenocarcinoma derivate from colon cancer (Caco-2 cell) using AgNOR staining methods in the literature. Therefore, we carried out the current study to show that there is an effect of capsaicin treatment for human colon adenocarcinoma derivate from colon cancer.

## 2. Materials and methods

### 2.1. Sample preparation

In this experimental study, the human colon adenocarcinoma derivate from colon cancer (Caco-2; HTB-37-American Type Culture Collection, Manassas, VA; #ATCC) cell line produced for commercial purpose was obtained from the manufacturer. The capsaicin solution was prepared with the dissolving of ground and bottled capsaicin specimen (Sigma) in 20 mL dimethyl sulfoxide (100% w/v) via a magnetic mixer during 1 day at 37 °C. Then the Caco-2 cells were cultured Dulbecco’s modified Eagles medium (DMEM F12; Sigma Chemical Co., St. Louis, MO, USA) including Streptomycin/Penicillin (100 U/ml; Sigma Chemical Co.) and 20% fetal calf serum (Sigma Chemical Co.) in a humidified atmosphere of 5% CO2 air at 37 °C. For the prevention of contamination risk, as quite as sterile conditions were performed. Then healthy Caco-2 cells were divided groups for capsaicin treatment. Tissue culture plate with 96 well including 100 μL of medium with 1 × 104 Caco cells was used to detect optimum capsaicin dose. These Caco-2 cells was cultured was in a humidified atmosphere 95% air containing 5% CO2 overnight to attach to the plate. Then the medium was removed and Caco-2 cells were rinsed via 200 μl phosphate-buffered saline (PBS) 3 times. The Caco-2 cells were identified. The study group was constituted as log concentrations of capsaicin (25uµ, 50 uµ, 75uµ) on colon cancer cells after the different cultured period. The capsaicin concentrations were implemented as dissolve in medium from stock concentration by half diluting to detected LD50 dose. Doses under the 25 micrograms have no effect on the cells.

### 2.2. Cytotoxicity in culture

Each group consisted of 3 wells. The detection of optimal doses was performed by viability test after cultured of the plate including the cells for 2 h at 37 °C in a 5% CO2 incubator. The Caco-2 cells treated with optimal Capsaicin concentration were used to detect the anticancer effect of capsaicin via the AgNOR technique. The AgNOR staining method was performed in 4 different groups.

### 2.3. AgNOR detection

**Figure 1:**
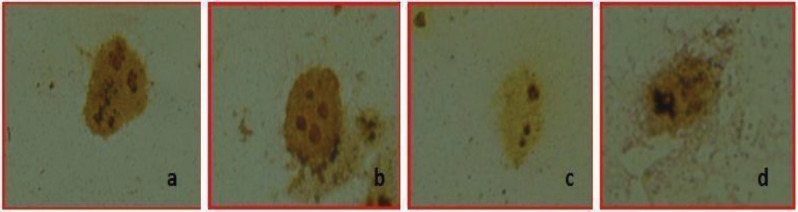
A demonstrative example of AgNOR staining of Caco-2 cells (a: control; b: 25 uμ capsaicin treated group; c: 50 uμ capsaicin treated group; d: 75 uμ capsaicin treated group).

Cultured Caco-2 cells with 25uµ, 50 uµ, 75 uµ capsaicin treated group and control group were spread on the clean slide and dried at room temperature. After air dried, the slides were fixed in fixative (3 volume methyl alcohol:1 volume acetic acid) and the AgNOR staining method was carried out according to the slight modification of the protocol followed by Benn and Perle [26] and Lindler [27]. The AgNOR stained slides were viewed with a light microscope (Eclipse 80i, Nikon, Tokyo, Japan) and photographed with a digital camera (Digital Sight DS-fi 1, Nikon). The captured images of Caco-2 cell were transferred to image processing software (ImageJ version 1.47t, National Institutes of Health, Bethesda, Maryland, USA) and both mean AgNOR number and total AgNOR area per nuclear area (TAA/NA) was calculated via “freehand selection” tool for each nucleus. Fifty nuclei were evaluated for each slide. A demonstrative example of AgNOR staining of Caco-2 cells was given in Figure 1 (a: positive control; b: 25 uµ capsaicin treated group; c: 50 uµ capsaicin treated group; d: 75 uµ capsaicin treated group). The mean AgNOR number and TAA/NA ratio of each group were given in Tables 1 and 2, respectively.

**Table 1:** TAA/NA and mean AgNOR number values of controls, capsaicin (25 uµ), capsaicin (50 uµ) and capsaicin (75 uµ) treated Caco-2 cells groups.

Groups	TAA/NA	Mean AgNOR number
Control-1 (n = 50)	0.178 ± 0.053	4.843 ± 1.321
Control-2 (n = 50)	0.175 ± 0.093	4.783 ± 1.217
Control-3 (n = 50)	0.179 ± 0.074	5.006 ± 0.974
Capsaicin (25 uµ)-1 (n = 50)	0.175 ± 0.104	4.696 ± 1.203
Capsaicin (25 uµ)-2 (n = 50)	0.173 ± 0.097	4.715 ± 1.084
Capsaicin (25 uµ)-3 (n = 50)	0.174 ± 0.086	4.784 ± 1.125
Capsaicin (50 uµ)-1 (n = 50)	0.159 ± 0.057	3.091 ± 1.312
Capsaicin (50 uµ)-2 (n = 50)	0.164 ± 0.071	3.389 ± 1.105
Capsaicin (50 uµ)-3 (n = 50)	0.160 ± 0.102	3.214 ± 1.218
Capsaicin (75 uµ)-1 (n = 50)	0.160 ± 0.025	3.109 ± 1.119
Capsaicin (75 uµ)-2 (n = 50)	0.158 ± 0.112	3.087 ± 1.105
Capsaicin (75 uµ)-3 (n = 50)	0.157 ± 0.091	3.112 ± 1.123

TAA/NA: Total AgNOR area/nuclear area, n: Number of measurements in each group.

**Table 2:** Double comparison of all groups for mean AgNOR number and TAA/Na ratio.

Groups	Average TAA/NA of groups	Mean AgNOR number	P value for TAA/NA and mean AgNOR number
Control (n = 50)	0.177 ± 0.002	4.877 ± 0.115	0.067* and 0.112*
Capsaicin (25 uµ) (n = 50)	0.174 ± 0.001	4.732 ± 0.046	0.001# and 0.000#
Capsaicin (50 uµ) (n = 50)	0.161 ± 0.003	3.231 ± 0.150	0.000& and 0.000&
Capsaicin (75 uµ) (n = 50)	0.158 ± 0.002	3.103 ± 0.137	0.205α and 0.212α

*: Pairwise comparison for control-capsaicin (25 uµ), #: Pairwise comparison for both of control-capsaicin (50 uµ) and Capsaicin (25 uµ)-Capsaicin (50 uµ), &: Pairwise comparison for both of control-capsaicin (75 uµ) and capsaicin (25 uµ)-capsaicin (75 uµ), α: Pairwise comparison for capsaicin (50 uµ)-capsaicin (75 uµ). TAA/NA: Total AgNOR area/nuclear area, n: Number of measurements in each group.

### 2.4. Statistical analysis

Statistical analyses were performed using the Statistical Package for Social Sciences (IBM Corp., Armonk, NY, USA) for Windows 22.0. The Shapiro-Wilk test was used to detection whether the data were normally distributed. Since we found that the data were normally distributed (P > 0.05), parametric tests were used for statistical analysis. The comparison of all groups was done using the 1-way ANOVA test. T-test was used for a pairwise comparison of all groups. Assumptions (normality and homogeneous variance) were investigated for 1-way ANOVA. Since the variances of the groups were homogeneous, Tukey test was performed. The results were given as mean ± SD, and P < 0.05 was considered to be statistically significant. 

## 3. Results

**Figure 2:**
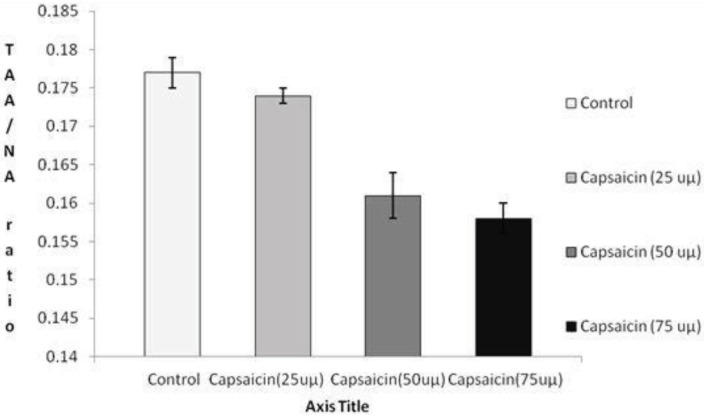
The comparison of all groups for both TAA/NA

**Figure 3:**
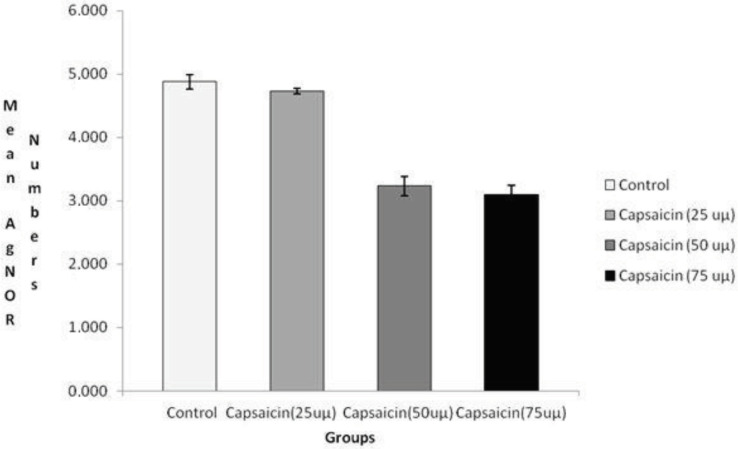
The comparison of all groups for mean AgNOR number.

When we compared all groups, statistically significant differences were detected among groups for both TAA/NA (P = 0.000) (Figure 2) and mean AgNOR number (P = 0.000) (Figure 3). Mean AgNOR number and TAA/NA ratio of positive control, capsaicin (25 uµ, 50 uµ, and 75 uµ) groups were given in Table 1. In the double comparison of the groups; while there were no significant differences between control and capsaicin (25 uµ) (P = 0.067) and between capsaicin (50 uµ) and capsaicin (75 uµ) (P = 0.205); significant differences were detected between positive control and capsaicin (50 uµ) (P = 0.001), between positive control and capsaicin (75 uµ) (P = 0.000), between capsaicin (25 uµ) and capsaicin (50 uµ) (P = 0.001) and between capsaicin (25 uµ) and capsaicin (75 uµ) (P = 0.000) for TAA/NA. When we were taken into consideration of mean AgNOR number, there were significant differences between positive control and capsaicin (50 uµ) (P = 0.001), between positive control and capsaicin (75 uµ) (P = 0.000), between capsaicin (25 uµ) and capsaicin (50 uµ) (P = 0.000) and between capsaicin (25 uµ) and capsaicin (75 uµ) (P = 0.000) for mean AgNOR number (Table 2).

## 4. Discussion

From the past to the present, phytochemical agents obtained from medicinal plants have been used for the treatment of human disorders. Description of new phytochemical agents and detection of the most reliable dose for cancer treatment are crucial to improving the diagnostic accuracy and management of the diseases. NORs are related to a majority of regulatory proteins and they have roles as functional subunits of the nucleolus [24]. Alterations of AgNOR protein amounts reflect the metabolic activities and protein synthesis capacity of the cells. Different studies are performed on malign and benign lesions [15–18]. Additionally, there are studies about the protective effects of phytochemical agents on cancer treatment and detection of the most reliable dose of these agents via AgNOR staining methods [21,22]. Despite the important advances for the management and treatment of the disease, the deaths depend on cancer have been increasing, too. Thus, different treatment strategies such as phytotherapy using natural therapeutic agents such as capsaicin may be used for disease treatment. Capsaicin had anticarcinogenic, antimutagenic, antigenotoxic and antiangiogenic effects [23]. In this study, we detected the antitumoral effect of capsaicin on human colon adenocarcinoma derivate from colon cancer. 

In this study, we detected that mean AgNOR number and TAA/NA ratio of positive control is significantly higher than the Caco-2 cell line treated with capsaicin (50 uµ and 75 uµ) groups for both mean AgNOR number and TAA/NA. Also, the Caco-2 cell line treated with capsaicin (25 uµ) had significantly higher mean AgNOR and TAA/NA ratio than the Caco-2 cell line treated with capsaicin (50 uµ and 75 uµ) groups (Table 2, Figures 2 and 3). The current study showed that when the capsaicin dose treatment increased, the mean AgNOR number and TAA/NA ratio decreased. In metabolically active cells, not only cellular morphology but also gene expression and its yields are altered. Calculating NOR protein expression gives information about the behavioral, metabolically activity and protein synthesis capacity of the cells [25]. Therefore, the detection of NOR proteins may be used to obtain knowledge about the anticancer effect of the phytotherapeutic agents and for the detection of reliable dose for therapeutic uses. According to the current study result, the most reliable dose of capsaicin is 50 uµ and 75 uµ. Due to the NOR proteins reflect the proliferation ratio of the cells, it may be said that the capsaicin has potentially cancer-protective effects.

To obtain more certain knowledge about the anticancer effects of the different phytotherapeutic agents such as capsaicin, additional studies related to different doses of the phytotherapeutic agents such as capsaicin are also needed.

## Disclaimers/Conflict of interest

There is no financial support in this study. The authors declare that they have no conflict of interest.

## Informed consent

In this study, cell lines were used. Since ethics committee approval is not required in cell line studies, there is no ethics committee approval date or number.
